# Supplementary material for: Longitudinal changes of iron in the substantia nigra of early-stage Parkinson’s disease
Source: medRxiv. 2026 Jun 3:2026.06.02.26354736. Preprint. [Version 1] doi: 10.64898/2026.06.02.26354736 (PMC13252487; doi:10.64898/2026.06.02.26354736)
Supplement: Supplementary file 1 [file media-1.pdf]

Supplemental Data for

Longitudinal changes of iron in the substantia nigra of early-stage Parkinson's disease

Du G, Sica C, De Jesus S, Kong L, Mailman RB, Huang X

Table of Contents

MRI acquisition and ROI segmentation. 2

Supplemental Figure 1. Definition of regions of interests (ROI) on T2w template image. 3

Supplemental Table 1. Longitudinal analysis results with linear mixed models. 4

Supplemental Table 2. Correlation between regional QSM values and clinical metrics at baseline. 5

Supplemental Table 3. Correlation between 1-year changes of regional QSM values and clinical metrics. 6

References: 7

MRI acquisition and ROI segmentation.

For each participant, T1-weighted, T2-weighted, and multi-gradient-echo MR images were acquired on a 3T Siemens PrismaFit scanner (Siemens Healthineers, Erlangen, Germany) with a 20-channel coil. The T1- and T2-weighted images are used as anatomical references. A magnetization-prepared rapid acquisition gradient echo sequence was used to obtain T1-weighted images with repetition time/echo time = 1540/2.34 milliseconds; field of view = 256×256; slice thickness, 1 mm (with no gap); and slice number = 176. A 3-dimensional T2-weighted SPACE (sampling perfection with application optimized contrast using different angle evolution) sequence was used to obtain T2-weighted images with repetition time/echo time = 2500/316 milliseconds and the same spatial resolution settings as the T1-weighted images. T2*-weighted images were acquired using a multi-gradient-echo sequence with 8 echoes (echo times ranging from 6.2 to 49.6 milliseconds); and repetition time = 55 milliseconds; flip angle = 15°; field of view = 240×240; matrix = 256×256; slice thickness = 2 mm; slice number = 64; and voxel size = $0.9\times0.9\times2$ mm^3. All images were inspected offline and deemed free of severe artifacts or any major structural abnormalities.

An atlas-based semiautomatic segmentation approach was used to segmented ROIs in the study. Namely, In step 1, T1- and T2-weighted images from all participants were used to construct a cohort-specific template using an unbiased atlas construction algorithm in the Advanced Normalization Tools (ANTS) package.[1,2] Since most of these structures have high iron content and are seen best in T2-weighted images, the ROIs were defined manually on the constructed T2-weighted template according to previous studies.[3,4] Supplemental Figure 1 shows the exact location of these five structures. In step 2, an atlas-based segmentation pipeline (AutoSeg v3.0; University of North Carolina Neuro Image Analysis Laboratory) generated the segmentation in individual space based on the T2-weighted images. In step 3, automatic segmentation results from all subjects were inspected visually and corrected manually by a rater blinded to group information. In step 4, an affine registration algorithm was used to bring the ROIs from T2-weighted images to the QSM images. Finally, mean QSM values from each ROI (including both left and right sides) were calculated for individual subjects.

Supplemental Figure 1. Definition of regions of interests (ROI) on T2w template image.

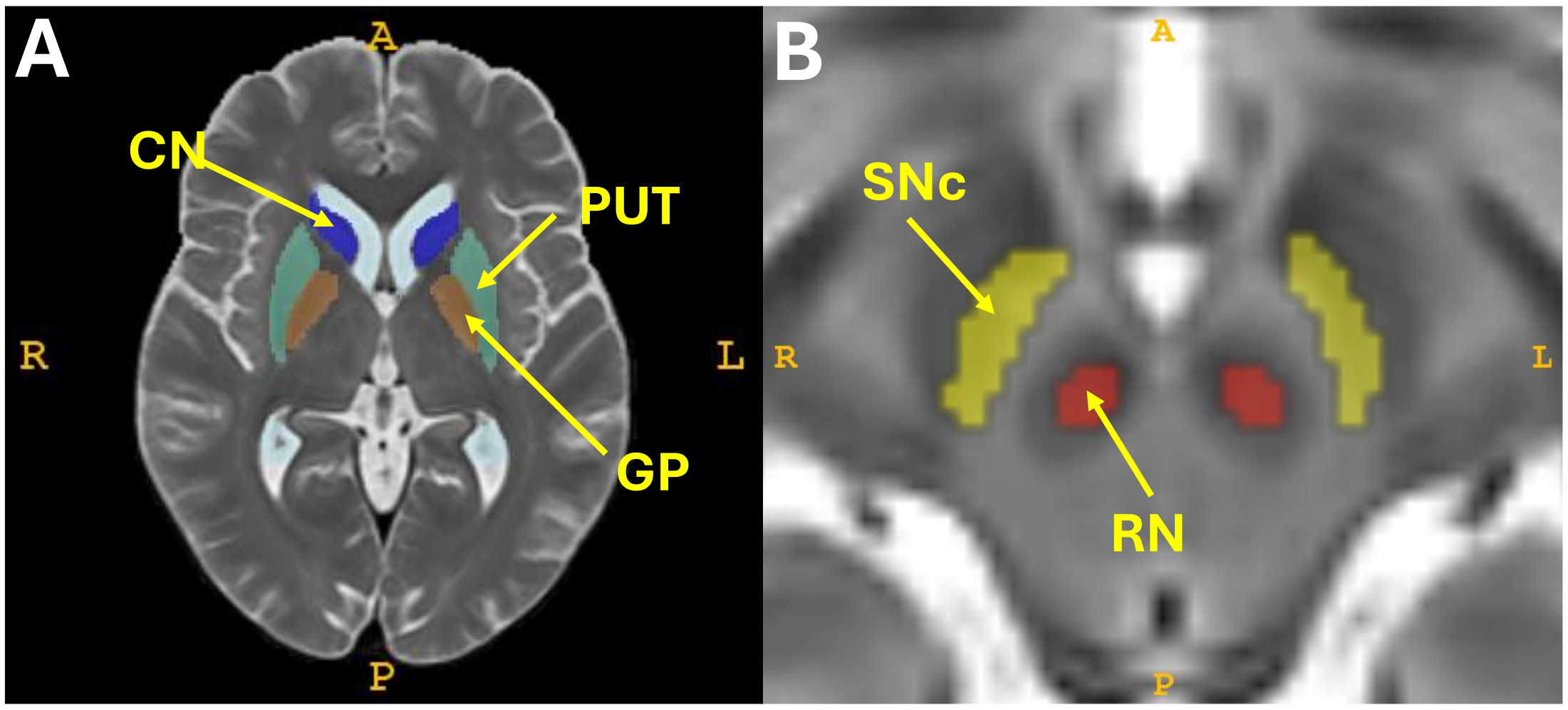

Supplemental Table 1. Longitudinal analysis results with linear mixed models.

	Visit	Group	Visit × Group	Age	Sex
Clinical metrics:					
MDS-UPDRS I	0.894	**0.0007***	0.567	0.675	0.926
MDS-UPDRS II	**0.032**	**<0.0001***	**0.046**	0.172	0.187
MDS-UPDRS III	0.270	**<0.0001***	0.328	**0.011**	0.365
GDS scores	0.625	**0.0063**	0.916	0.805	0.259
MoCA scores	0.767	**0.018**	0.079	**0.026**	0.916
QSM measures:					
SNc	**0.0028***	**0.0008***	**0.0036***	0.741	0.259
RN	0.470	0.092	0.402	0.206	0.737
PUT	0.626	0.159	0.619	**0.013**	0.822
CN	0.840	0.182	0.760	0.470	0.420
GP	0.916	0.114	0.271	0.989	0.108

The p-values are from mixed effects models. * indicates significance after Bonferroni correction.

Supplemental Table 2. Correlation between regional QSM values and clinical metrics at baseline.

		**SNc**	**RN**	**PUT**	**CN**	**GP**
**MDS-UPDRS I**	r	0.268	0.105	0.234	***0.447***	0.124
	p	0.176	0.602	0.241	***0.019***	0.538
**MDS-UPDRS II**	r	-0.072	-0.067	0.302	***0.423***	-0.149
	p	0.722	0.741	0.126	***0.028***	0.458
**MDS-UPDRS III**	r	0.139	0.093	-0.101	***0.408***	0.247
	p	0.489	0.646	0.615	***0.034***	0.213
**GDS score**	r	0.186	0.043	0.176	0.130	0.125
	p	0.352	0.830	0.380	0.519	0.536
**MoCA score**	r	-0.140	-0.060	-0.199	-0.189	***-0.392***
	p	0.486	0.764	0.320	0.344	***0.043***

Supplemental Table 3. Correlation between 1-year changes of regional QSM values and clinical metrics.

		SNc	RN	PUT	CN	GP
**MDS-UPDRS I**	r	***0.462***	0.256	0.229	0.303	0.233
	p	***0.023***	0.227	0.282	0.150	0.273
**MDS-UPDRS II**	r	***0.536***	0.290	0.193	0.154	0.042
	p	***0.005****	0.159	0.356	0.463	0.842
**MDS-UPDRS III**	r	0.242	0.047	-0.238	-0.188	-0.061
	p	0.244	0.822	0.251	0.368	0.773
**GDS score**	r	-0.024	-0.134	***0.566***	0.351	-0.255
	p	0.911	0.523	***0.003****	0.085	0.219
**MoCA score**	r	0.141	0.232	-0.029	0.003	-0.011
	p	0.501	0.266	0.891	0.989	0.958

The p-values are from partial Pearson correlation analyses. * indicates significance after Bonferroni correction.

References:

1 Avants BB, Tustison NJ, Song G, Cook PA, Klein A & Gee JC (2011) A reproducible evaluation of ANTs similarity metric performance in brain image registration. *Neuroimage* **54**, 2033-2044.'doi':10.1016/j.neuroimage.2010.09.025.PMC3065962

2 Avants BB, Epstein CL, Grossman M & Gee JC (2008) Symmetric diffeomorphic image registration with cross-correlation: evaluating automated labeling of elderly and neurodegenerative brain. *Med. Image Anal* **12**, 26-41.'doi':S1361-8415(07)00060-6 [pii];10.1016/j.media.2007.06.004 [doi]

3 Wang J, Vachet C, Rumple A, Gouttard S, Ouziel C, Perrot E, Du G, Huang X, Gerig G & Styner M (2014) Multi-atlas segmentation of subcortical brain structures via the AutoSeg software pipeline. *Front Neuroinform* **8**, 7.'doi':10.3389/fninf.2014.00007.PMC3915103

4 Du G, Lewis MM, Sica C, He L, Connor JR, Kong L, Mailman RB & Huang X (2018) Distinct progression pattern of susceptibility MRI in the substantia nigra of Parkinson's patients. *Mov Disord* **33**, 1423-1431.'doi':10.1002/mds.27318.PMC6185755
